# Mutations of Glu560 within HIV-1 Envelope Glycoprotein N-terminal heptad repeat region contribute to resistance to peptide inhibitors of virus entry

**DOI:** 10.1186/s12977-019-0496-8

**Published:** 2019-12-03

**Authors:** Chen Yuan, Jia-Ye Wang, Hai-Jiao Zhao, Yan Li, Di Li, Hong Ling, Min Zhuang

**Affiliations:** 1 Department of Microbiology, Harbin Medical University, Harbin, Heilongjiang China; 2 Heilongjiang Provincial Key Laboratory of Infection and Immunity, Harbin, China; 3 Key Laboratory of Pathogen Biology, Harbin, China; 4 Wu Lien-Teh Institute, Harbin Medical University, Harbin, China

**Keywords:** Fusion inhibitor, HIV-1, Resistance, Mutation of Glu560, Virus entry, Conformational changes, gp41

## Abstract

**Background:**

Peptides corresponding to N- and C-terminal heptad repeat regions (HR1 and HR2, respectively) of gp41 can inhibit HIV-1 infection in a dominant negative manner by interfering with refolding of the viral HR1 and HR2 to form a six-helix bundle (6HB) that induces fusion between viral and host cell membranes. Previously, we found that HIV-1 acquired the mutations of Glu560 (E560) in HR1 of envelope (Env) to escape peptide inhibitors. The present study aimed to elucidate the critical role of position 560 in the virus entry and potential resistance mechanisms.

**Results:**

The Glu560Lys/Asp/Gly (E560K/D/G) mutations in HR1 of gp41 that are selected under the pressure of N- and C-peptide inhibitors modified its molecular interactions with HR2 to change 6HB stability and peptide inhibitor binding. E560K mutation increased 6HB thermostability and resulted in resistance to N peptide inhibitors, but E560G or E560D as compensatory mutations destabilized the 6HB to reduce inhibitor binding and resulted in increased resistance to C peptide inhibitor, T20. Significantly, the neutralizing activities of all mutants to soluble CD4 and broadly neutralizing antibodies targeting membrane proximal external region, 2F5 and 4E10 were improved, indicating the mutations of E560 could regulate Env conformations through cross interactions with gp120 or gp41. The molecular modeling analysis of E560K/D/G mutants suggested that position 560 might interact with the residues within two potentially flexible topological layer 1 and layer 2 in the gp120 inner domain to apparently affect the CD4 utilization. The E560K/D/G mutations changed its interactions with Gln650 (Q650) in HR2 to contribute to the resistance of peptide inhibitors.

**Conclusions:**

These findings identify the contributions of mutations of E560K/D/G in the highly conserved gp41 and highlight Env’s high degree of plasticity for virus entry and inhibitor design.

## Background

The HIV-1 envelope glycoprotein (Env) forms a trimer of noncovalently associated heterodimer of gp120 and gp41. The binding of the surface subunit gp120 to the CD4 receptor on host cells triggers conformational changes of Env that allow gp120 binding to the chemokine receptor CCR5 or CXCR4. After that, the hydrophobic fusion peptide at N-terminal region of transmembrane subunit gp41 inserts into cell membrane and the pre-hairpin intermediate forms [1–4]. Subsequently, three C-terminal heptad-repeat region (HR2) helices refold and package in the grooves of coiled-coil core assembled by N-terminal heptad-repeat region (HR1) in an antiparallel manner and a thermostable six-helix bundle (6HB) forms [5, 6]. This process drives target cell membrane and viral membrane closely and facilitates fusion pore formation to allow viral core to release into cytoplasm [7].

The fusion process provides possibilities to block virus entry and prevent HIV-1 infection. The first fusion inhibitor licensed for clinical use, enfuvirtide (also referred to as T20 or Fuzeon) that mimics HR2 sequences of gp41 can package with the coiled coil core of HR1 in an antiparallel manner to form a heterogenous 6HB (inhibitor bundle) that interferes with the formation of the homogenous 6HB (endogenous bundle) [8–14]. However, the problem of virus resistance to T20 is still a big challenge. The clinical and laboratory studies reported a bunch of databases of viruses which were resistant to T20 [13, 15–19]. The common escape mechanism is mutations within HR1 that reduce binding activity of T20 to the hydrophobic groove of the HR1 coiled coil. Meanwhile, additional adaptive mutations in HR2 can compensate the change of HR1 residues to form more stable endogenous bundle or facilitate fitness of viruses [20–24].

Peptides that mimic HR1 segment also inhibit virus fusion, but they are not yet in clinical use [25]. The lower solubility than HR2 peptide and the tendency to aggregate in solution [26–28] reduce the effective concentration and potency of HR1 inhibitors. Two manners associated with the inhibitory mechanism have been reported. The first one is that HR1 peptide inhibitors automatically form into trimers and interact with HR2 to interrupt the formation of the endogenous 6HB; the other one is that HR1 peptide inhibitors interfere with the formation of the internal coiled-coil core of HR1 [29]. HIV-1 can also generate resistance to N peptide inhibitors, but the resistance mutations can stabilize the formation of 6HB, unlike the situation of C peptide inhibitor [30–33].

The reported resistance mutations to T20 were consistent in vitro and in vivo. The highest frequency of mutations for T20 was within the region of amino acids 547–556 of Env [15, 34]. Except for above 10 amino acids in the key region for resistance to T20, the other mutations in HR1 and HR2 were also reported [35, 36]. Among them, a glutamic acid to glycine substitution, a glutamic acid to aspartic acid substitution and a glutamic acid to lysine substitution at position 560 (E560G, E560D, and E560K, respectively) in HR1 (according to HXB2 numbering) were found from a patient under T20 therapy [16, 36]. The former two substitutions (E560G and E560D) were also found in cell cultures under the selection pressure of T20 in vitro (our unpublished data) and one mutation E560K was found from resistance cultures to N peptide inhibitors [29, 37], respectively. Interestingly, the resistance mutations occurred under N peptide inhibitor N36 and N44 selections by two pathways, either E560K mutation in HR1 or a glutamic acid to lysine substitution at position 648 (E648K) in HR2 [29, 37, 38]. And the E560K substitution was also found under IZN36 peptide selection [29, 37]. These results lead us to focus on the effects of the position 560 on resistance to fusion inhibitors and virus entry.

To investigate the property and resistance mechanisms of these substitutions at position 560 in HR1, we constructed HIV-1 LAI pseudoviruses bearing Env with mutations E560K/D/G. The mutation E560K conferred resistance to N36 and IZN36 through increasing endogenous 6HB stability, but E560G or E560D as compensatory mutations destabilized the 6HB to reduce inhibitor binding and resulted in increased resistance to T20. The sensitivity of all mutants to soluble CD4 (sCD4) and the broadly neutralizing Abs (bNAbs) targeting membrane proximal external region (MPER), 2F5 and 4E10, was enhanced. The molecular modeling analysis also revealed the possible conformational changes of the E560K/D/G mutations indicating that the residue at the position 560 could regulate Env conformations through interactions with gp120 and HR2 of gp41. Our findings further clarified the molecular mechanism for the resistance mutations at position 560 to fusion peptide inhibitors and provided new insights for HIV-1 entry and inhibitor design.

## Results

### Effects of Mutations E560K/D/G on HR1 peptide resistance

Envs with the mutations at position 560 were incorporated into pseudoviruses for assessing their contributions to resistance. The infectivity of pseudoviruses bearing Env with E560K, E560D or E560G was lower in U87CD4^+^CXCR4^+^ cells that express high levels of CD4 and CXCR4 receptors (Additional file 1: Fig. S1). Pseudoviruses with mutation E560K or E560D had higher infectivity but pseudovirus with mutation E560G had lower infectivity compared to that of the wild type in RC4 cells that express lower levels of CD4 receptors (Additional file 1: Fig. S1), implicating both mutants E560K and E560D may be also responsible for adaptation to the low levels of CD4 receptors which were used for selections of all resistance viruses.

**Fig. 1:**
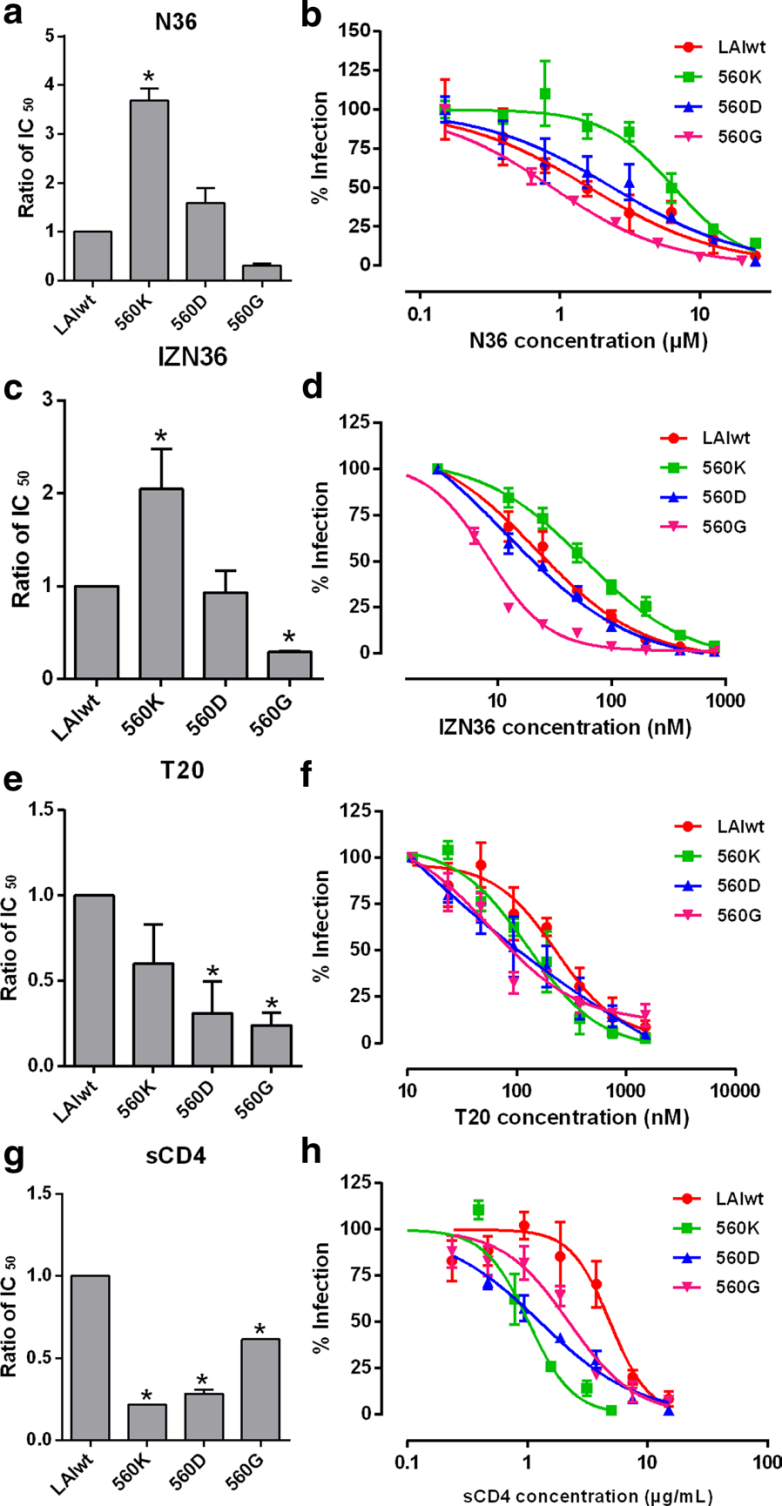
Peptide and sCD4 inhibition of pseudovirus bearing Envs with E560K/D/G mutations. The IC_50_ values for the N36, IZN36, T20 and sCD4 were determined for pseudoviruses with Envs containing various mutations in RC4 cells, normalized to the IC_50_ for the wild-type pseudovirus (LAIwt). Averages and SD values (error bars) of at least three independent experiments are shown. The susceptibility and dose-dependent curves of pseudoviruses with indicated mutation to N36 (**a**, **b**), IZN36 (**c**, **d**), T20 (**e**, **f**) and sCD4 (**g**, **h**) are shown. *p < 0.05 compared with LAIwt

The sensitivity of HIV-1 LAI wild type and mutants with E560K, E560D or E560G to HR1 peptide inhibitors were detected in RC4 cells. The mutation E560K conferred resistance to N36 and IZN36 and the IC_50_ values to both peptides were higher than that of wild type, while mutants with E560D and E560G were similar or more sensitive to N36 and IZN36 (Table 1, Fig. 1a, c). The dose-dependent manner of inhibitory activity of N36 and IZN36 was shown (Fig. 1b, d). It is consistent with our previous finding that the E560K mutation derived from HIV-1 under selections of N peptide inhibitors in vitro [37].

**Table 1:** Inhibition by HR1 and HR2 peptide inhibitors and sCD4

Inhibitor selection	Mutants	N36 (μM)		IZN36 (nM)		T20 (nM)		sCD4 (μg/mL)	
		IC_50_ ± SD	Ratio	IC_50_ ± SD	Ratio	IC_50_ ± SD	Ratio	IC_50_ ± SD	Ratio
N36/IZN36	LAIwt	1.77 ± 0.22	1.00	29.3 ± 8.95	1.00	251.14 ± 59.67	1.00	4.85 ± 0.015	1.00
	E560K	6.52 ± 0.43	3.68	59.86 ± 12.93	2.04	148.89 ± 58.78	0.60	1.05 ± 0.010	0.21
T20	E560D	2.81 ± 0.55	1.58	23.15 ± 1.24	0.93	135.92 ± 36.69	0.54	1.37 ± 0.10	0.28
	E560G	0.55 ± 0.07	0.31	11.22 ± 0.90	0.29	87.66 ± 21.48	0.24	2.98 ± 0.010	0.61

The ratio is IC_50_ for the indicated pseudovirus/LAIwt pseudovirus

### Mutations E560K/D/G alter sensitivity to inhibition by T20 and sCD4

Because the LAI Envs with E560D/G mutations emerged under T20 selection in vitro (our unpublished data) and three mutations E560K/D/G were reported under treatment of T20 in clinical data [36], the sensitivity of all mutants to T20 was examined (Fig. 1e, f). Three mutants showed more sensitive to T20 in RC4 cells than that of wild type. It raised the possibility that these mutations are assistant mutations and could strengthen the effect of resistance when the founding resistance mutations such as L544S and G546D are present (Table 2). Therefore, we analyzed the T20-selected clones containing substitutions L544S, G547D, and E560G/D which appeared in above order in the selected cultures in vitro (our unpublished data). Interestingly, we found that only mutant E560G showed dramatic sensitivity to T20, while IC_50_ values of the mutants with E560G and either L544S or G547D increased by 21.73- and 12.68-folds, respectively. Surprisingly, the mutant containing L544S, G547D, E560G and the mutant containing L544S, G547D, E560D dramatically increased resistance activity with IC_50_ values enhanced by 555.16- and 224.18-folds (Table 2). These results indicated that mutations of E560G/D assisted the founding resistance mutations and enhanced resistance activity to T20.

**Table 2:** Inhibition of pseudoviruses with resistance mutations by HR2 peptide inhibitor, T20

	T20 (nM) IC_50_ ± SD	Ratio	*p* value
LAIwt	138.34 ± 39.45	1.00	
L544S	8248 ± 682.35	59.62	< 0.001
G547D	1749.02 ± 233.44	12.64	< 0.001
E560G	61.71 ± 11.71	0.45	0.0057
L544S G547D	25,310.03 ± 2985.82	182.96	< 0.001
L544S E560G	3006.20 ± 548.26	21.73	< 0.001
G547D E560G	1753.74 ± 340.63	12.68	0.0022
L544S G547D E560G	> 76,800	> 555.16	< 0.001
L544S G547D E560D	31,012.25 ± 3364.73	224.18	< 0.001

The ratio is IC_50_ for the indicated pseudovirus/LAIwt pseudovirus

Next, we investigated whether these mutations altered the sensitivity to sCD4. All three mutants increased sensitivity by 1.63- to 4.62-folds to sCD4 compared to wild type, suggesting that the specific residues at the position 560 could influence the interaction between Env and CD4 receptors (Table 1, Fig. 1g, h).

### Mutations E560K/D/G confer sensitivity to bNAbs 2F5 and 4E10

**Fig. 2:**
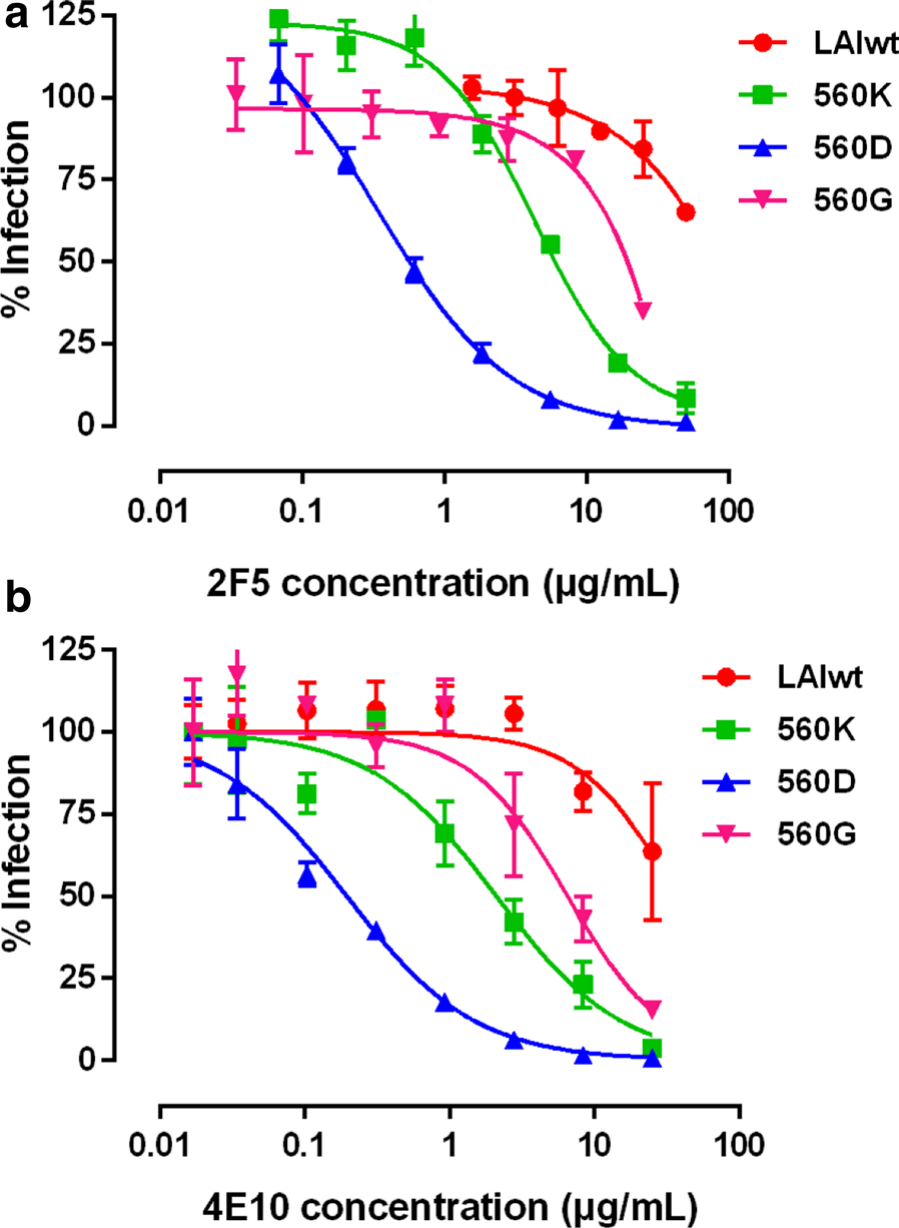
Inhibition of pseudoviruses bearing Envs with E560K/D/G mutations by broad neutralizing antibodies, 2F5 and 4E10. The IC_50_ values for the 2F5 and 4E10 targeting at MPER of gp41 were determined for pseudoviruses with Envs containing indicated mutations in U87CD4^+^CXCR4^+^ cells, normalized to the IC_50_ for the wild-type pseudovirus (LAIwt). Averages and SD values (error bars) of at least three independent experiments are shown. The susceptibility and dose-dependent curves of pseudoviruses with indicated mutation to 2F5 (**a**) and 4E10 (**b**) are shown. *p < 0.05 compared with LAIwt

The bNAbs 2F5 and 4E10 targeting MPER of gp41 can neutralize diverse cross-clade primary isolates at 67% and 100% [5, 39–41]. 2F5 recognizes linear epitope (ELDKWA) and blocks virus access to the target cells by inhibiting the formation of gp41 6HB structure. The 4E10 recognizes C-terminus of 2F5 epitope (NWFDIT), and this recognition interferes with the formation and expansion of MPER intervals [42]. In order to verify whether these mutations at position 560 of Env affect the MPER exposure in the virus fusion process and sequentially impact virus entry, we further examined the sensitivity of the mutants to 2F5 and 4E10 (Table 3, Fig. 2). The results showed that the neutralization sensitivity of the mutants with E560K/D/G to 2F5 and 4E10 enhanced. IC_50_ values of the mutants with E560K/D/G generally reduced approximately 2.81- to 37.31-folds to 2F5 and 3.78- to 147.06-folds to 4E10, respectively, compared to that of wild type. Based on these data, we speculated that the mutations E560K/D/G would alter Env conformations and lead to better exposure of MPER or extend the exposure time of gp41 MPER during virus entry.

**Table 3:** Susceptibilities of pseudoviruses containing E560K/D/G mutations to the broadly neutralizing Abs 2F5 and 4E10

Inhibitor selection	Mutants	2F5 (μg/mL)		4E10 (μg/mL)	
		IC_50_ ± SD	Ratio	IC_50_ ± SD	Ratio
N36/IZN36	LAIwt	> 50	1.00	> 25	1.00
	E560K	6.26 ± 0.53	< 0.13	2.24 ± 0.153	< 0.073
T20	E560D	1.34 ± 2.52	< 0.027	0.17 ± 0.01	< 0.054
	E560G	17.78 ± 0.10	< 0.36	6.61 ± 0.26	< 0.21

The ratio is IC_50_ for the indicated pseudovirus/LAIwt pseudovirus

### Effect of mutations on HR1-HR2 Interactions

**Fig. 3:**
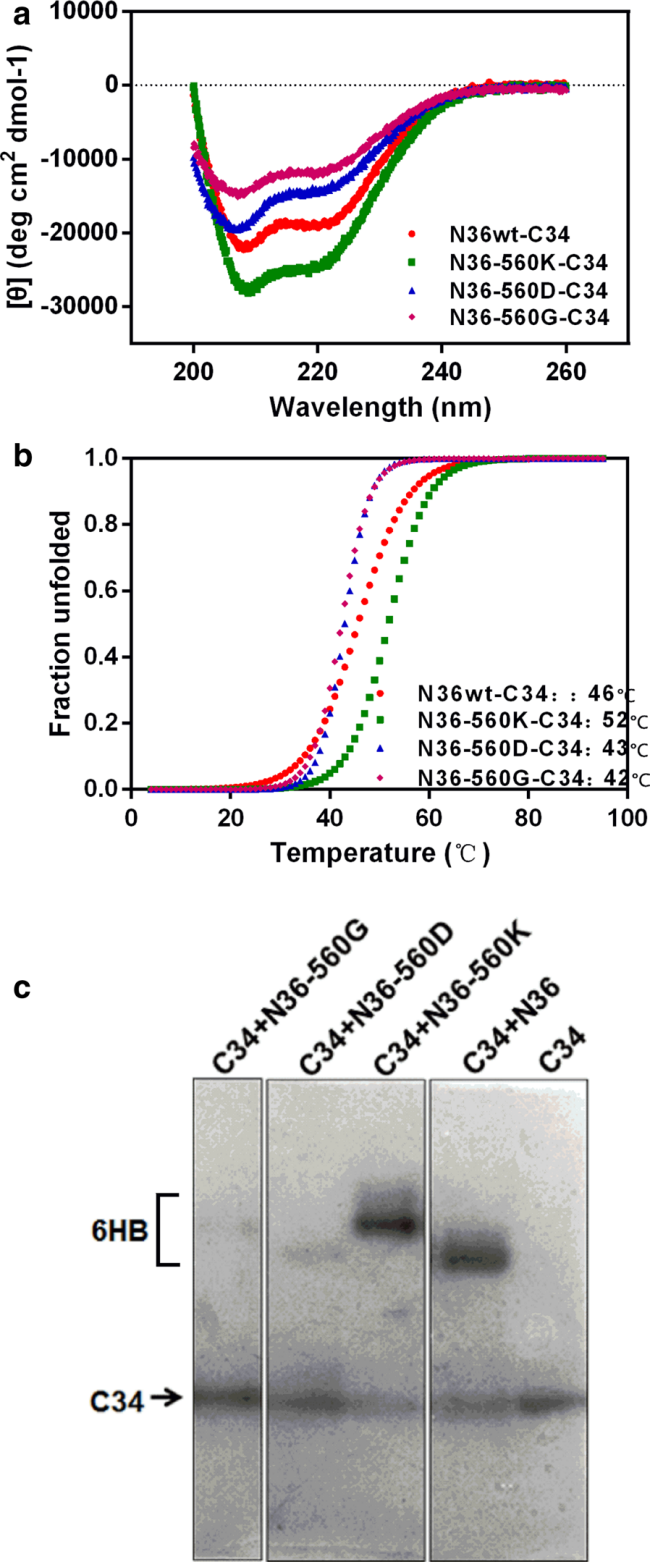
Biophysical characterizations of six-helix bundle (6HB) formed by N36 with indicated mutations and C34. The α-helical content is calculated from the circular dichroism (CD) spectroscopy signal at the indicated wavelengths. Unfolding is recorded at 222 nm by CD spectroscopy at the indicated temperatures, with calculated transition midpoints (*Tm* values) shown. The CD scanning of the complexes formed by N36 with indicated mutations and the C34 peptide (**a**) and their melting curves (**b**) are shown. **c** The 6HB formed by N36 with indicated mutations and C34 are visualized using native PAGE electrophoresis. The upward migration of the bands represents 6HB and lower bands represent C34 peptide. The bands of 6HB formed by C34 and N36 E560K or E560G migrated upward due to reduced negative charges of 6HB. Three panels are from the same gel, but lanes with irrelevant peptides are removed

Mutations in the HR1 would not only directly interfere with inhibitor binding to gp41 but also probably affect the affinity of endogenous 6HBs. We detected the effects of these mutations at the position 560 of 6HB structure using circular dichroism and native PAGE. The α-helicity and thermal stability of 6HB formed by the interaction of C34 with N36 or its mutants at equimolar concentration were analyzed (Table 4, Fig. 3a, b). The 6HB formed by C34 and the wild-type N36 and all mutants demonstrated high helical content (Fig. 3a, c). The helicity formed by C34 and each N36 E560D or E560G was lower than that of wild-type N36 (Fig. 3a, c). The bands of 6HB formed by C34 and N36 E560K or E560G migrated upward due to reduced negative charges (Fig. 3c). The N36 and its mutants migrated off the gel due to the net positive charge, thus only the C34 and complex of 6HB were showed here. All mixtures of C34 and N36 or its mutants displayed relatively high transition midpoints (*Tm*) in their thermal denaturation curves (ranging from 42 °C to 52 °C), consistent with the formation of 6HB structures (Table 4; Fig. 3b). Significantly, each mutation in HR1 changed the *Tm* of peptide mixtures that represented the endogenous bundle when inhibition of N peptide inhibitors occurred or that represented the both endogenous and inhibitor bundles when inhibition of HR2 peptide inhibitors occurred, because the mutation only exists in the virus but not in the inhibitors. Analysis on the thermal stability of 6HB showed that E560K increased the *Tm* (∆*Tm*) by 6 °C, but mutation E560D or E560G decreased the *Tm* (∆*Tm*) by 3 °C or 4 °C, respectively, compared to wild-type N36.

**Table 4:** Thermal denaturation studies of the six-helix bundles formed by mixtures of HR1 (N36) and HR2 (C34) peptides with or without resistance mutations

	N36-C34	
	*Tm* (°C)	∆*Tm* (°C)
N36wt	46	–
N36-E560K	52	6
N36-E560D	43	− 3
N36-E560G	42	− 4

*Tm* midpoint of the thermal unfolding transition; *ΔTm* change in Tm due to the mutation compared with the peptide without mutation

## Discussion

Entry inhibitors are considered as potential drugs for the treatment of HIV-1 infection, particularly in patients with viruses resistant to reverse transcriptase and protease inhibitors. T20, which is HR2 mimic of gp41 can be used as fusion inhibitor on the all disease stages and is effective for both CCR5 and CXCR4 tropic viruses. Resistance to T20 is conferred by critical mutations in the HR1 region of gp41. And N peptide fusion inhibitors are HR1 mimics of gp41 including N36 and IZN36. These HR1 peptide inhibitors were designed by Kim et al. and composed of N36 peptide and leucine zipper in the N terminus of N36 [6]. IZN36 has greater water solubility and can maintain a stable coiled-coil trimer which solves the problem of N peptide aggregation and results in significant increasing of its antiviral activity. To date HR1 peptide inhibitors are not yet approved for treatment of HIV-1 infection and information about their inhibitory mechanisms and resistance data are limited.

In our previous work the mutations E560D and E560G were found in the resistance LAI strains under T20 selection in vitro by increasing its concentration gradually (our unpublished data). And E560K is observed in the screened HIV-1 JRcsf [29] and LAI [37] resistance strains to N36 and IZN36 in vitro. Therefore, we thought that the position 560 which is exposed outside of the 6HB core might play a crucial role on resistance to peptide inhibitors. According to the alignment of Env sequences from Los Alamos HIV database (https://www.hiv.lanl.gov/content/index), the major residue at position 560 is glutamic acid (E), and other residues such as aspartic acid (D), lysine (K) and glycine (G) are also observed in HIV-1 primary isolates. These data indicate that although the residue at the position 560 in the Env of HIV-1 strains is highly conserved, various mutants exist in nature.

**Fig. 4:**
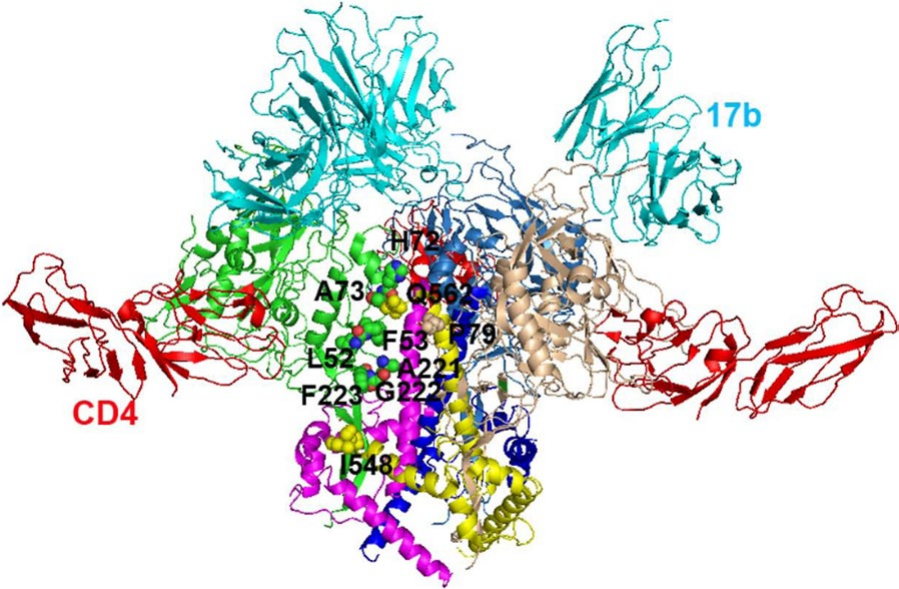
Mutations of Glutamic acid 560 (E560) in the HR1 modeled in Env trimers. The supposed residues which are associated with HR1 are modeled in the CD4- and 17b-liganded Env SOSIP trimer (PDB code: 5VN3). The CD4 molecules are colored red and 17b are colored cyan. Each protomer of gp120 and gp41 are colored green and purple, blue and skyblue, and wheat and yellow, respectively. The residues in gp120 are colored green or wheat, and the residues in gp41 are colored yellow. The view of the topological layers in the gp120 inner domain and gp41 HR1 region highlight the residues in gp120 that located in and around its potential interaction sites with the residue at position 560 of gp41. The following residues belong to different layers: L52, F53, H72, A73, and P79 in layer 1; A221, G222, and F223 in layer 2. The residues between I548 and Q562 are not observed in this model

Based on these data, we generated the pseudoviruses with wild-type or mutant Env proteins and assessed infectivity and neutralization activity by inhibitors. The pseudoviruses bearing Env containing E560K or E560D mutation showed higher infectivity in the RC4 cells than wild type (Additional file 1: Fig. S1) indicating these mutations may be also responsible for adaptation to the PM1 cell line which is used for selection of resistance strains in vitro and expressed low level of CD4 receptors [29, 37]. The mutant containing E560G substitution had lower infectivity in both U87CD4^+^CXCR4^+^ and RC4 cells than wild type. Meanwhile, the sensitivity of these three mutants to sCD4 increased (Table 1 and Fig. 1g, h), suggesting that the mutations E560K and E560D conferred the virus to sufficiently utilize the CD4 receptor on the target cells with lower levels of CD4 receptors and may result in conformational changes to affect the binding of viral envelope protein gp120 to CD4 receptors indirectly. To gain insight into how the mutations affected the sensitivity of Env to peptide inhibitors and sCD4, we predicted atomic interactions in the adjacent regions of the E560K, E560G, and E560D mutations using PYMOL software. The structure analysis of mutants revealed that the residues at the position 560 may affect the interactions with the residues surrounding H72, F53 and A221 in the inter molecule gp120 or with the residue P79 surrounding residues in the intra molecule gp120 and further change the binding to CD4 receptor (PDB code: 5VN3) (Fig. 4) [43, 44]. Although there is not showing the residue at position 560 in this structure model (5VN3), the suspected location of the position 560 could be between I548 and Q562. The residues H72, F53 and P79 are involving in topological layer 1 of gp120 inner domain, which are resided near the trimeric axis of the Env glycoprotein complex and would be well positioned to interact with gp41 [43]. The residue A221 which belongs to layer 2 of gp120 inner domain may also interact with the residue at the position 560. These two regions as two potentially flexible topological layer 1-layer 2 in the gp120 inner domain apparently contribute to the interaction with gp41 in the unliganded Env trimer [43]. The changes of mentioned above residues including F53, H72, P79 and A221 apparently decreased the gp120-gp41 association implicating that some critical interactions existed between them and HR1 of gp41 and further affect the CD4 binding or off rate of the bound CD4.

Although the correlations between faster entry kinetics and decreased sensitivity to peptide fusion inhibitors have been reported [45], in the previous study we found that the resistance to N-peptide fusion inhibitors correlated with thermodynamic stability of the gp41 six-helix bundle but not faster HIV-1 entry kinetics [46]. The present study showed that mutations at position 560 (E560K/D/G) conferred sensitivity to the bNAbs targeting MPER (Table 3, Fig. 2). The bNAb 2F5 binds to both native and fusion-intermediate conformations and is supposed to inhibit a relatively late step in virus entry [47]. Both Abs 2F5 and 4E10 recognize a linear epitope within MPER with high affinity [48, 49]. The increasing sensitivity to these bNAbs may be explained that the three mutants, especially E560D and E560K, promoted more exposure of neutralizing epitopes within MPER or extend exposure time of these epitopes indirectly.

The Glutamic acid (E) at the position 560 is the only negative charged residue in the N36 region and occupy position b in the 4,3-hydrophobic heptad repeat which oriented into outside of 6HB. The substitution of a positively charged residue Lysine (E560K), a negative charged residue Aspartic acid (E560D), or an uncharged residue Glycine (E560G) changed the size of side chain or charges and may disrupt the interactions between HR1 with HR2 or gp120. These changes may involve in the resistance mechanisms to fusion peptide inhibitors. The one universal mechanism of improving the stability of the endogenous 6HB to favor the virus might be a more efficient resistance mechanism, especially for an inhibitor that can bind to two different sites of Env.

**Fig. 5:**
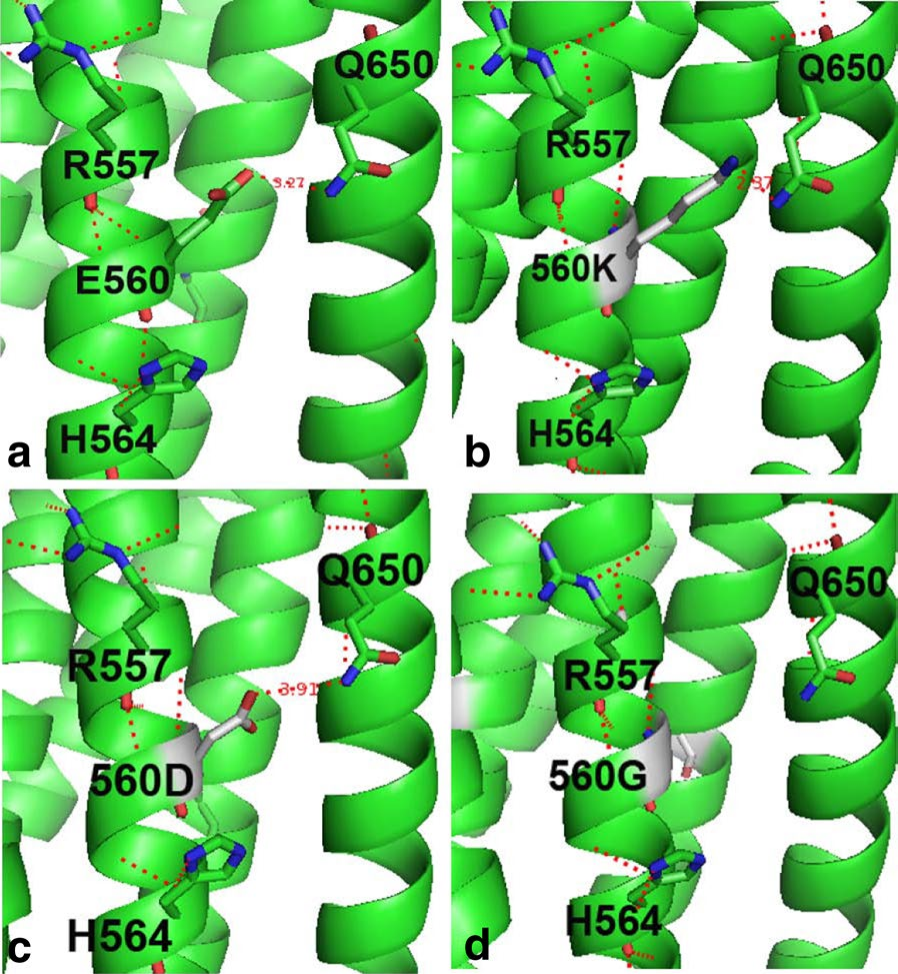
Modeling the mutations of Glutamic acid 560 (E560) in the six-helix bundle (6HB). The wild-type E560 and mutations E560K/D/G are modeled in the 6HB conformation (PDB code: 1AIK) in a ribbon model in longitudinal view. **a** E560 residue interacts with Q650 in C34 of inter-protomer gp41 via hydrogen bond which has 3.27 Å distances between two residues. **b** The mutation E560K changed into the positively charged residue and has longer side chain than wild type, and it still interacts with Q650 with shorter distance between them (2.37 Å), or probably it forms two hydrogen bonds with nitrogen atom of Q650. **c** E560D mutation has only shorter side chain than wild type and may interrupt the hydrogen bond with the longer distance (3.91 Å) between E560D and Q650. **d** E560G mutation removes the interaction between E560 and Q650. Interatomic distances are marked by the *dashed lines*

In the present study the results showed that compared with wild-type pseudovirus, E560K mutant was resistant to N36 and IZN36 and was slightly sensitive to T20, E560D mutant showed similar IC_50_ values to N36 and IZN36 and was sensitive to T20, and E560G mutant was sensitive to all tested fusion peptide inhibitors (Table 1, Fig. 1). In agreement with results of the CD analyses, 6HB formation by N36 or its mutant and C34 was observed in the native PAGE studies (Fig. 3a, c). The higher *Tm* of 6HB containing E560K mutation from CD analysis may let virus form more stable endogenous bundle and be resistant to N peptide inhibitors, and it was also suspected to bind to T20 more stably. The structure analysis revealed that E560 interacts with Q650 in HR2 of the intra protomer with the hydrogen bond which has 3.27 Å distances between two residues (Fig. 5a) (PDB code: 1AIK) [50]. E560K mutation still interacts with Q650 through hydrogen bond with shorter distance between them (2.37 Å), or probably its two hydrogen atoms forms two hydrogen bonds with nitrogen atom of Q650. Therefore, this interaction may strengthen the stability of 6HB (Fig. 5b). E560D mutation carries similar negative charge as wild type, but it has only shorter side chain than wild type and may interrupt the hydrogen bond with the longer distance (3.91 Å) between E560D and Q650 (Fig. 5c) that may be responsible for the reducing stability of 6HB by 3 °C. The 6HB containing E560G mutation is less stable than that containing wild type by 4 °C and it can be explained by that the interaction between E560 and Q650 is interrupted (Fig. 5d). E560D and E560G mutants showed less binding to HR2 but were still not resistant to T20 suggesting they are compensatory mutations with the major resistance mutations L544S and G547D.

The major resistance mechanisms to T20 are considered as the less binding of T20 to HR1, steric obstruction, electrostatic attraction and electrostatic repulsion [51]. It was reported that the LLSGIV stretch of the N-terminal region of HIV-1 gp41 is critical for binding to T20 [52]. We found that resistance mutants containing L544S, G547D and E560G/D mutations in the selection of T20 in vitro (our unpublished paper). The susceptibility analysis of the mutants harboring the separated mutations suggested that E560G mutation dramatically increased the resistance activity to T20 compared to the mutants with two mutations L544S and G547D (Table 2). In high-resolution structures (PDB code: 1ENV) [53], L544 residue faces to hydrophobic cleft formed by the interface of the HR1 trimeric coiled coil, including the hydrophobic residues L545, L548 and V549 (Additional file 1: Fig. S2a, b). Mutation L544S removes the interactions between these hydrophobic residues and result in the destabilization of 6HB, possibly contributing to the resistance to T20 (Additional file 1: Fig. S2c). The 547D is negative charged residue which may interact with E659 residue through electrostatic repulsion to interrupt its binding to HR2 of the inter-helical protomer gp41 or peptide inhibitor T20 (Additional file 1: Fig. S2d). Meanwhile, we also observed the lower *Tm* (∆Tm: − 7 °C) of 6HB containing G547D mutation compared to that containing wild type based on CD analysis (our unpublished data). And as the analyzing above, E560D and E560G mutations reduced the stability of 6HB through rearrangements of hydrogen bonds between them and Q650 network (Additional file 1: Fig. S2e). Therefore, we supposed that these three resistance mutations may have synergistic effects for destabilization of 6HB and result in more than 555.16-fold increase of resistance to T20.

## Conclusion

In summary, these results indicate that forcing HIV to escape peptide inhibitors targeting Env fusion-intermediates reveal different mechanisms of resistance to N and C peptide inhibitors, but select mutations at same position 560 of Env. HIV-1 selects E560K mutation to increase thermostability of the 6HB and is further resistant to N peptide inhibitors, and selects E560G or E560D as compensatory mutations to destabilize the 6HB to reduce inhibitor binding and to be resistant to T20. These findings highlight Env’s high degree of plasticity and suggest that the different residues at position 560 of HIV-1 Env can regulate Env conformational changes through cross interaction to gp120 and gp41 to affect the binding of CD4, neutralizing Abs targeting MPER and inhibition by fusion peptide inhibitors.

## Methods

### Cells and plasmids

U87 cells expressing CD4 and CXCR4 (U87CD4^+^CXCR4^+^) were kindly provided by Dan Littman (New York University). HEK 293T cells were purchased from America ATCC cell bank. RC4 cells that are derived from HeLa cells and express low levels of CD4 and CCR5 were a gift from David Kabat (Oregon Health and Science University, Portland, OR) [54]. The expression vector pCMV/R, the Env-deficient HIV-1 genome plasmid pCMVΔ8.2 and the pHR’-Luc that contains the reporter gene were provided by Gary Nabel (National Institutes of Health, Bethesda, MD).

### Reagents

Peptides LAI N36 (corresponding to HXB2 residues 546–581; SGIVQQQNNLLRAIEAQQHLLQLTVWGIKQLQARIL), LAI N36 560K (SGIVQQQNNLLRAIKAQQHLLQLTVWGIKQLQARIL), LAI N36 560D (SGIVQQQNNLLRAIDAQQHLLQLTVWGIKQLQARIL), LAI N36 560G (SGIVQQQNNLLRAIGAQQHLLQLTVWGIKQLQARIL), LAI IZN36 (IKKEIEAIKKEQEAIKKKIEAIEKEISGIVQQQNNLLRAIEAQQHLLQLTVWGIKQLQARIL), LAI T20 (HXB2 residues 638–673; YTSLIHSLIEESQNQQEKNEQELLELDKWASLWNWF) and LAI C34 (HXB2 residues 628–661; WMEWDREINNYTSLIHSLIEESQNQQEKNEQELL) were synthesized by Shanghai JiEr biochemistry company. SDS-PAGE and analytic high performance liquid chromatography (HPLC) showed that the purity of all peptides were over 95%. The molecular weight of all peptides was confirmed by matrix-assisted laser desorption ionization-time-of-flight mass spectroscopy (MALDI-TOF–MS). The bNAbs 2F5 and 4E10 were provided by NIH (NIH AIDS Reagent Program, Division of AIDS, NIAID, NIH). Soluble CD4 (sCD4) was purchased from Progenics Pharmaceuticals (Tarrytown, NY).

### Site-directed mutagenesis

Env-expressing plasmids pCMV/R-Env with E560K, E560D or E560G were created by overlapped PCR using pCMV/R-Env(LAI) wild type (Genebank No. K02013.1) [55] as a template and two pairs of primers containing desired mutations (Additional file 2: Table S1). PCR was performed using high-fidelity thermostable polymerase PrimeSTAR (Takara, Dalian, China). The PCR products were digested by the restriction enzymes NotI and EcoR V and replaced the wild-type *env* gene within pCMV/R-Env(LAI). Each plasmid was confirmed by sequencing the full-length *env* gene.

### Pseudovirus inhibitory assay

HIV-1 pseudoviruses were generated by the cotransfection with 0.5 µg of the Env-expressing plasmid, 4 µg of Env-deficient HIV-1 genome plasmid (pCMVΔ8.2) and 4 µg of reporter plasmid (pHR’-Luc) in 5 × 10^6^ 293T cells per 10 cm-diameter dish. Supernatants were collected and filtered at 48 h after transfection and quantified by a p24 enzyme-linked immunosorbent assay (ELISA) (ZyptoMetrix, Buffalo, NY). Pseudoviruses containing 5 ng of p24 infected target cells U87CD4^+^CXCR4^+^ and RC4 cells (2 × 10^4^ cells/well in 96-well plates), and their infectivity was normalized to wild type. The infectivity of serially diluted pseudoviruses was also detected and 50% tissue culture infectivity dose (TCID_50_) was calculated. The equivalent TCID_50_ inocula of pseudoviruses was added to each well of target cells (2 × 10^4^ cells/well in 96-well plates) in the presence of peptide, sCD4 or bNAbs in a total volume of 100 µl supplemented with 2.5 µg/ml of DEAE (Sigma). At 48 h postinfection, the luciferase activity was measured using luciferase substrate (Promega, WI) on a Modulus™II microplate multimode reader (Turner Biosystems, Promega, WI).

### Circular dichroism spectroscopy

HR1 and HR2 peptides were mixed (10 µM each) in 50 mM sodium phosphate (pH 7.0) containing 150 mM NaCl and incubated at 37 °C for 30 min (final volume, 0.2 ml). These peptides or peptide mixtures were acquired on a Chirascan spectropolarimeter (Applied Photophysics, UK) at 20 °C using a 1.0 nm bandwidth, 0.1 nm resolution, 0.1 cm pathlength, 4.0 s response time, and a 5 nm/min scanning speed. The spectrums were corrected by subtraction of a blank corresponding to the solvent [19, 56]. Thermal denaturation was monitored at 222 nm by applying a thermal gradient of 2 °C/min in the range of 4 °C to 95 °C. Reverse melt from 95 to 4 °C was also detected. The melting curve was smoothed, and the midpoint of the thermal unfolding transition (T*m*) value was determined using Chirascan software. The T*m* averages of at least two measurements for each complex were calculated.

### Native PAGE

The 6HB which is composed of HR1 and HR2 peptides was detected by native PAGE as described previously [57]. The peptide pairs of C34 and N36 of each variant were incubated at a final concentration of 40 μM at 37 °C for 30 min. These mixtures were loaded onto an 18% Tris–glycine gel. The gel was then stained with Coomassie Blue and imaged using GIS-2010 system (Tanon, China).

### Statistical analysis

The 50% inhibitory concentration (IC_50_ value) of peptide inhibitors for each pseudovirus was calculated by nonlinear regression analysis using GraphPad Prism software (La Jolla, CA). The average IC_50_ for each mutant or inhibitor was compared to that for the wild-type pseudovirus using student *t* test with non-parametric assumption and *p* values equal to or less than 0.05 were considered significant.

## Supplementary information

**Additional file 1: Figure S1.** Infectivity and western blot of pseudoviruses bearing Env with mutations E560K/D/G. (a) Infectivity of pseudoviruses in U87CD4^+^CXCR4^+^ and RC4 cells. The relative luciferase infectivity is indicating that the ratio is IC_50_ for the indicated pseudovirus/LAIwt pseudovirus. The averages and SD values (*error bars*) of at least three independent experiments are shown. *p < 0.05 compared with LAIwt. (b) Western blot of pseudovirions bearing the envelope glycoproteins with mutations of E560. Top panel shows a blot probed with a monoclonal antibody (Chessie 8, AIDS reference and Reagent Program, NIH) recognizing gp160 and gp41. Bottom panel shows the same blot probed with a monoclonal antibody VAK4 [58] recognizing p24. **Figure S2.** Modeling the mutations of L544S, G547D and E560/D/G in the six-helix bundle (6HB). Mutation L544S is modeled in the 6HB conformation (PDB entry 1ENV) in a ribbon model in cross-section view (a) and longitudinal view (b). The 6HB of wild type (c), the mutants with mutations L544S, G547D and E560G (d) or with mutations L544S, G547D and E560D (e) are modeled. Interatomic distances are marked by the *dashed lines*.

**Additional file 2: Table S1.** Primer sequences for LAI mutant env creation.

## Data Availability

All data generated or analyzed during this study are included in this published article and its additional files.

## Abbreviations

Env: envelope protein
gp41: HIV envelope transmembrane subunit
gp120: HIV envelope surface subunit
HR1: heptad-repeat region one or N-terminal heptad repeat
HR2: heptad-repeat region two or C-terminal heptad repeat
6HB: six-helix bundle
sCD4: soluble CD4
bNAbs: broadly neutralizing antibodies
MPER: membrane proximal external region
ELISA: enzyme linked immunosorbent assay
CD: circular dichroism
*Tm*: transition mid-point temperature
