# Supplementary material for: Mutations of Glu560 within HIV-1 Envelope Glycoprotein N-terminal heptad repeat region contribute to resistance to peptide inhibitors of virus entry
Source: Retrovirology. 2019 Dec 3;16:36. doi: 10.1186/s12977-019-0496-8 (PMC6889725; doi:10.1186/s12977-019-0496-8)
Supplement: Supplementary file 1 — Additional file 1: Figure S1. Infectivity and western blot of pseudoviruses bearing Env with mutations E560K/D/G. (a) Infectivity of pseudoviruses in U87CD4+CXCR4+ and RC4 cells. The relative luciferase infectivity is indicating that the ratio is IC50 for the indicated pseudovirus/LAIwt pseudovirus. The averages and SD values (error bars) of at least three independent experiments are shown. *p < 0.05 compared with LAIwt. (b) Western blot of pseudovirions bearing the envelope glycoproteins with mutations of E560. Top panel shows a blot probed with a monoclonal antibody (Chessie 8, AIDS reference and Reagent Program, NIH) recognizing gp160 and gp41. Bottom panel shows the same blot probed with a monoclonal antibody VAK4 [58] recognizing p24. Figure S2. Modeling the mutations of L544S, G547D and E560/D/G in the six-helix bundle (6HB). Mutation L544S is modeled in the 6HB conformation (PDB entry 1ENV) in a ribbon model in cross-section view (a) and longitudinal view (b). The 6HB of wild type (c), the mutants with mutations L544S, G547D and E560G (d) or with mutations L544S, G547D and E560D (e) are modeled. Interatomic distances are marked by the dashed lines. [file 12977_2019_496_MOESM1_ESM.pdf]

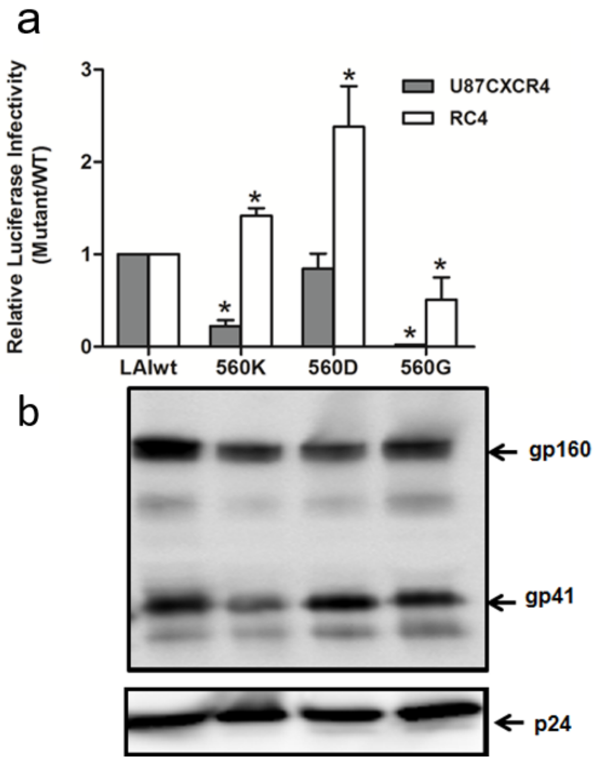


**Figure S1. Infectivity and western blot of pseudoviruses bearing Env with mutations E560K/D/G.** (a) Infectivity of pseudoviruses in U87CD4$^{+}$CXCR4$^{+}$ and RC4 cells. The relative luciferase infectivity is indicating that the ratio is $IC_{50}$ for the indicated pseudovirus/LAIwt pseudovirus. The averages and SD values (*error bars*) of at least three independent experiments are shown. *, $p < 0.05$ compared with LAIwt. (b) Western blot of pseudovirions bearing the envelope glycoproteins with mutations of E560. Top panel shows a blot probed with a monoclonal antibody (Chessie 8, AIDS reference and Reagent Program, NIH) recognizing gp160 and gp41. Bottom panel shows the same blot probed with a monoclonal antibody VAK4 recognizing p24.

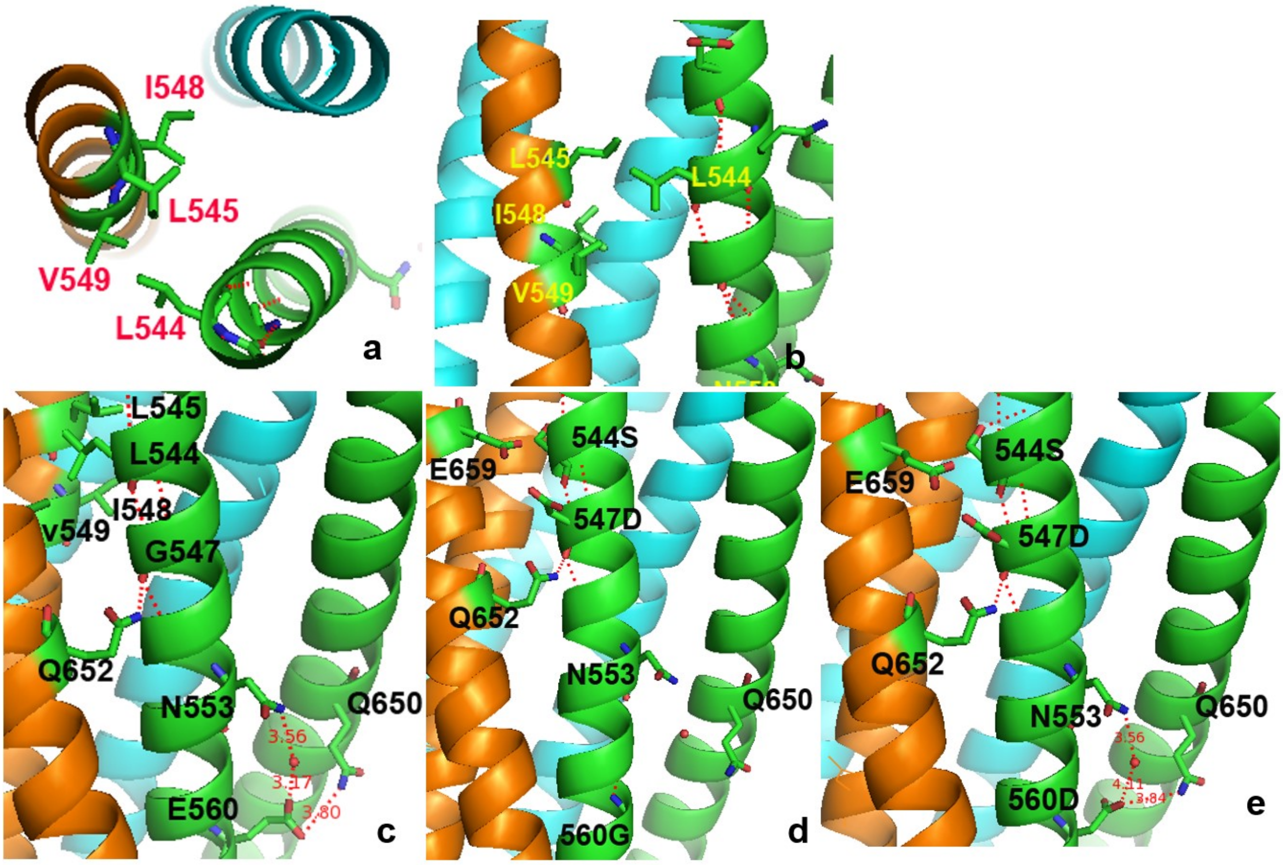


**Figure S2. Modeling the mutations of L544S, G547D and E560/D/G in the six-helix bundle (6HB)**. Mutation L544S is modeled in the 6HB conformation (PDB entry 1ENV) in a ribbon model in cross-section view (a) and longitudinal view (b). The 6HB of wild type (c), the mutants with mutations L544S, G547D and E560G (d) or with mutations L544S, G547D and E560D (e) are modeled. Interatomic distances are marked by the *dashed lines*.
