# Supplementary material for: Mutations of Glu560 within HIV-1 Envelope Glycoprotein N-terminal heptad repeat region contribute to resistance to peptide inhibitors of virus entry
Source: Retrovirology. 2019 Dec 3;16:36. doi: 10.1186/s12977-019-0496-8 (PMC6889725; doi:10.1186/s12977-019-0496-8)
Supplement: Supplementary file 2 — Additional file 2: Table S1. Primer sequences for LAI mutant env creation. [file 12977_2019_496_MOESM2_ESM.pdf]

Table S1. Primer sequences for LAI mutant env creation

Primers	Sequences
**LAI wt forward**	5-TATCC***GATATC***GCCGCCACCATGAGAGTGAAGGAGAAATATC-3
**LAI wt reverse**	5-TCTAGA***GCGGCC***GCTTATAGCAAAATCCTTTCCAAGC-3
**E560G forward**	5-AATTTGCTGAGGGCTATT<u>GGA</u>GCTCAACAGCATCTGTTGCAAC-3
**E560G reverse**	5-GTTGCAACAGATGCTGTTGAGCTCCAATAGCCCTCAGCAAATT-3
**E560D forward**	5-AATTTGCTGAGGGCTATT<u>GAC</u>GCGCAACAGCATCTGTTGCAAC-3
**E560D reverse**	5-GTTGCAACAGATGCTGTTGCGCGTCAATAGCCCTCAGCAAATT-3
**E560K forward**	5-GCTATT<u>AAG</u>GCGCAACAGCATCTGTTGCAACTCAC-3
**E560K reverse**	5-GCGCCTTAATAGCCCTCAGCAAATTGTTCTG-3
